# The Role of Executive Functioning and Academic Achievement in the Academic Self-Concept of Children and Adolescents Referred for Neuropsychological Assessment

**DOI:** 10.3390/children5070083

**Published:** 2018-06-21

**Authors:** Brittany A. Bailey, Sophia K. Andrzejewski, Sarah M. Greif, Adrian M. Svingos, Shelley C. Heaton

**Affiliations:** Department of Clinical and Health Psychology, University of Florida, Gainesville, FL 32610, USA; sandrzej@ufl.edu (S.K.A.); sgreif1021@ufl.edu (S.M.G.); adrianmchambers@phhp.ufl.edu (A.M.S.); sheaton@ufl.edu (S.C.H.)

**Keywords:** executive function, self-concept, academic achievement

## Abstract

The current study evaluated a model of youth academic self-concept which incorporates practical executive functioning behaviors and academic achievement. Though greater academic achievement has been linked to both positive self-concept and better executive functioning, these constructs have not been examined simultaneously. It was hypothesized that academic achievement would mediate the association between problems with executive functioning and academic self-concept such that youth with more problems with executive functioning would have lower academic achievement and, in turn, lower academic self-concept. Clinical data was analyzed from a diagnostically heterogeneous sample of youth (*n* = 122) who underwent neuropsychological evaluation. Problems with executive functioning were assessed using the Behavior Rating Inventory of Executive Function. Academic achievement was assessed using the Woodcock–Johnson Tests of Achievement or Wechsler Individual Achievement Test. Academic self-concept was assessed using the youth-report version of the Behavioral Assessment System for Children. Surprisingly, findings indicate that academic achievement is not significantly associated with problems with executive functioning or academic self-concept. However, greater problems with executive functioning are associated with decreased academic self-concept. The overall model included several covariates and accounted for 10% of the variance in academic self-concept. Findings suggest that executive skills may be essential for aligning academic achievement with classroom performance. Though various child characteristic covariates were included, the model accounted for a small amount of variance suggesting that future studies should examine contributing contextual factors.

## 1. Introduction

Low self-concept is associated with a number of unfavorable social and emotional outcomes. Previous research indicates that academic achievement is associated with youth self-concept. Academic achievement, in turn, is often influenced by executive dysfunction, a cognitive impairment observed in a number of pediatric populations. Despite well-established connections between these variables, little research has examined the associations between executive functioning, academic achievement, and self-concept concurrently, and no known studies have explored these relationships simultaneously in pediatric populations characterized by executive dysfunction. The examination of these constructs simultaneously may provide a foundation for research further clarifying contributing factors to the development of low self-concept and examining additional avenues for intervention.

### 1.1. Self-Concept: Development and the Role of Academic Achievement

Self-concept, or the internal evaluation of one’s own strengths and weaknesses, is an important contributing factor to a wide range of important life outcomes. Low self-concept has been implicated in internalizing problems, such as depressed affect and feelings of hopelessness [1]. There is also evidence that lower self-concept may be related to externalizing problems, such as aggression, antisocial behavior, and delinquency [2]. Thus, understanding factors of interest as they relate to adaptive levels of self-concept remains an important area of continued study.

Self-concept is conceptualized as a multidimensional construct. Global self-concept is the culmination of interrelated dimensions, with perceived strengths and weaknesses in dimensions that are more important to the individual contributing more [3]. In early childhood, youth are typically unable to understand or verbalize global self-concept. However, measures of dimension-specific self-concept at this age often indicate undifferentiated and unrealistically high self-perceptions [4]. In middle and late childhood, as children begin to gain the cognitive capacity to differentiate between their ideal and real performance, to utilize social comparison in personal evaluations, and to more accurately judge what other people are thinking, self-representations become more accurate and global self-concept may decline [5]. Thus, assessment of self-concept may have the most clinical utility if conducted during or after middle childhood.

Research has consistently found a positive relationship between academic achievement and self-concept, suggesting academic achievement is an important factor influencing self-concept. Specifically, self-concept has been correlated with academic achievement in a large sample of sixth graders, such that one standard deviation higher academic self-concept was associated with greater academic skill mastery equivalent to having received three extra months of academic instruction [6]. This association is likely bidirectional. That is, youth who perform poorly in school develop negative perceptions of their academic performance. In turn, youth with negative perceptions of their academic performance have lower motivation and a decreased tolerance for frustration, which further impacts their achievement in the classroom.

Research in children with specific learning disorders also suggests that socio-economic status (SES) may play a role in the relationship between academic skills and self-concept. For instance, a study by Smith et al. in 1997 found that children with learning disorders from higher SES homes reported lower self-concept than children from lower SES homes. As a possible explanation for this finding, the authors suggest that parents of higher SES may have greater expectations of their children’s academic performance than parents of lower SES [6].

Unfortunately, the practice of retaining students who may not be ready to transition to new grade levels has a negative impact on students’ self-concept. Studies indicate that youth who are retained early in their academic careers exhibit lower self-concept than same grade peers years following their retention [7]. Given that retention by nature involves overt judgement of a child’s performance relative to his peers, these findings are not surprising. However, they do indicate that prior grade retention should be taken into consideration when examining youth’s self-concept in the academic context.

There is also evidence that gender is an important factor to consider when assessing academic self-concept as it relates to academic skills. Previous research suggests that girls tend to attribute their academic accomplishments to external causes rather than their own competency [8]. Further, when compared to boys who perform similarly academically, girls report lower academic self-concept [9]. These studies suggest that academic achievement may have a differential effect on academic self-concept in female and male youth.

### 1.2. Executive Functioning: Development and Association with Academic Achievement

Though many operational definitions of executive functioning exist throughout the research literature and a prevailing definition has yet to be elucidated, the current study defines executive functions as a set of “self-directed actions needed to choose goals and to create, enact, and sustain actions toward those goals [10,11,12,13]”. These actions include emotion regulation or control over emotional responses and impulse control or inhibition, as well as problem-solving skills, such as independent initiation of problem solving, planning, organization, progress monitoring, and set shifting [14]. In contrast to other cognitive domains that reach maturity during childhood, executive functioning follows a protracted developmental course. That is, though executive skill development begins in infancy, the developmental rate accelerates in late childhood through adolescence, only to reach mature levels in early adulthood [15].

Executive functioning delays and deficits are common across a wide range of pediatric clinical populations and typically associated with frontal network dysfunction. This dysfunction may occur either developmentally, such as in Attention-Deficit/Hyperactivity Disorder (ADHD) and other neurodevelopmental disorders, or may occur as the result of a neurological insult, such as a traumatic brain injury or the delivery of cancer-treating agents that affect the central nervous system [10,16,17].

Owing in part to its multidimensional nature, measurement of executive functioning has been approached in a variety of ways. Measurement falls into two broad categories: performance-based tests and informant ratings. Performance-based assessment of executive functioning often occurs in a one-on-one setting with fewer distractions than the typical environment. Questionnaires assessing “practical executive functioning behaviors” incorporate items capturing executive skills occurring in the context of everyday activities in a naturalistic environment. Ratings may be obtained directly from the youth in question, or from a caregiver (i.e., parent, legal guardian) or teacher. Interestingly, research suggests that executive functioning correlates poorly across performance-based and rating scales such that these assessment methods may in fact be tapping into different aspects of this construct and have different utility [18]. In their review of measures of executive functioning, Toplak, West, and Stanovich suggest that, because performance-based measures are administered in optimal performance conditions, they assess the efficiency of an individual’s goal pursuit while rating scales assess the success of goal pursuit under typical conditions [19].

Previous research has consistently found significant positive associations between performance-based executive functioning and youth academic achievement [20]. For example, Bull and Scerif identified significant associations between performance-based measures of attention shifting, working memory, and inhibition and performance-based tests of mathematical achievement in a large sample of third grade students [21]. A study conducted by Latzman, Elkovitch, Young, and Clark found that cognitive flexibility and monitoring test performance was positively associated with reading achievement and inhibition test performance was positively associated with mathematics achievement in adolescents [22]. Additionally, in a clinical sample of seven to fifteen year-olds with ADHD, a performance-based measure of attention shifting was found to be associated with academic achievement across domains [23]. In regards to classroom performance, prior research also suggests that youth with ADHD and clinically significant executive skill deficits are up to twice as likely to be retained as their peers with ADHD but without these deficits [24]. Overall, research strongly supports positive associations between performance-based measures of executive functioning and academic achievement.

However, far fewer studies have explored this relationship using informant reports of practical executive functioning behaviors in the context of everyday activities. Studies which have examined this association have largely focused on the children’s emotion regulation in their academic achievement. Previous research has indicated significant associations between emotion regulation in early elementary school and performance-based mathematics and literacy achievement [25]. In middle school, ratings of emotion regulation have been positively associated with grade point average [26]. The association between more global measures of practical executive functioning behaviors and academic achievement remains unclear.

### 1.3. Executive Functioning and Academic Self-Concept

The link between self-concept and academic achievement is well-established. As the preceding section highlights, executive functioning is positively associated with academic achievement. Thus, it stands to reason that executive functioning may also be positively associated (either directly or indirectly) with academic self-concept. At this time, only one known study has examined these variables simultaneously. Conducted by Roebers, Cimeli, Röthlisberger, and Neuenschwander [27], this study longitudinally examined the impact of performance-based executive functioning and academic self-concept on academic achievement in first (at Time One) and second graders (at Time Two). Though this study contributed to the literature by indicating that executive functioning is associated with perception of academic competence in childhood, it included academic achievement as an outcome without examining any possible mediating role it may play in the association between executive functioning and academic self-concept. Additionally, the participants were very young. As previously discussed, executive functions develop significantly in middle to late childhood through adolescence and this age coincides with a developmentally typical decline in self-concept. Thus, exploration of these associations in older youth may significantly expand upon these findings.

Logically, it stands to reason that if executive dysfunction is associated with poorer academic achievement, which is in turn associated with low academic self-concept, executive dysfunction would also be associated with lower academic self-concept. However, there is also reason to believe that poor executive functioning may be protective against low self-concept. For instance, inflated self-concept has been reported in youth diagnosed with ADHD compared to same aged-peers [28]. One possible explanation for this inflated self-concept is the presence of ADHD-related executive dysfunction which prevented these youth from reflecting on their own behavior and how it compares to the ideal [29]. These opposing possibilities underscore the necessity of further research to examine associations between executive dysfunction and aspects of self-concept.

Previous research suggests there is a complex relationship between youth academic achievement, self-concept, and executive functioning that will be important to better understand before effective interventions can be implemented to produce desired outcomes. Despite evidence that executive dysfunction is common across a wide variety of pediatric clinical populations and research highlighting deleterious outcomes associated with poor self-concept, no studies have directly examined the relationship between these variables in youth who (1) have reached an age at which typically developing children can report their self-concept; and (2) exhibit high rates of executive dysfunction. Additionally, prior studies examining associations between executive functioning and academic achievement have primarily used performance-based measures of executive functioning, leaving the question of how measures of practical executive functioning behaviors may be related to academic achievement.

The goal of the current study was to examine the associations between practical executive functioning behaviors, academic achievement, and academic self-concept in a clinical pediatric population. It was hypothesized that greater problems with executive functioning would be related to more problems with academic self-concept and that this relationship will be mediated by academic achievement.

## 2. Materials and Methods

Participant data was extracted from an Institutional Review Board (IRB)-approved databank of patients referred for neuropsychological testing at a University-affiliated psychology clinic. Participants were included if they completed all of the necessary measures for the current study and were between the ages of 8 years, 0 months and 17 years, 11 months at the time of evaluation. Participants were excluded if they had received a diagnosis of Autism Spectrum Disorder (ASD), as it is currently unclear how deficits in social reciprocity may impact the development of self-concept.

One hundred and twenty-two participants met the study’s inclusion criteria. The majority of participants were male (62.3%) and White (83.1%). Consistent with the population typically seen in these settings, participants exhibited a broad range of comorbid diagnoses. Thus, only primary diagnoses were included in analyses. The primary diagnosis was assigned under the supervision of the licensed assessing clinician and reflected the most impairing diagnosis assigned to the child in the context of the assessment, with the majority of participants receiving a primary diagnosis of ADHD (55%) and all participants receiving at least one diagnosis. Though the sample included a full range of SES, participants were on average middle class. Additional sample characteristics are depicted in Table 1 and Table 2.

Caregivers completed a demographic form which collected information regarding the caregiver’s relationship to the child, the child’s age, gender, race, and ethnicity, and whether there was a history of grade retentions. This form also included details about the caregiver’s educational attainment and current occupation which was used in an algorithm to calculate an index of SES as described by Hollingshead, with lower index scores reflecting higher levels of SES [30].

Academic self-concept was assessed using the Sense of Inadequacy subscale of the Behavioral Assessment System for Children, Self-Report of Personality, Child and Adolescent versions, 2nd Edition (BASC-2), a measure which demonstrates strong psychometric properties and has been externally validated [31]. The Sense of Inadequacy subscale is a 12-item scale assessing the child’s “perceptions of being unsuccessful in school, unable to achieve one’s goals, and generally inadequate”. It is composed of five true or false items and seven items rated on a four-point Likert scale regarding how frequently that item was experienced over the past several months (e.g., “I am disappointed with my grades” rated from “0” (never) to “3” (frequently)). Higher scores on this measure indicate greater difficulty with academic self-concept. Age- and gender-based normative-scores were used for all analyses as this was the scoring method employed when the data was collected for clinic use. T-scores greater than 60 represent an area of “Clinically Significant” concern and/or difficulty [31].

The current study sought to identify difficulties that may relate to executive dysfunction in everyday life. Thus, the Behavioral Rating Inventory of Executive Functioning (BRIEF), a parent-report measure of youth executive functioning with strong psychometric properties, was selected [14]. The BRIEF is comprised of 86 items which contribute to eight subscales and three global composite scores. This measure also yields a Global Executive Composite (GEC), (a composite of all measured domains), which was utilized in the current study. As BRIEF items center on problems with executive functioning, higher scores indicate greater levels of dysfunction. All analyses utilized norm-referenced, age-based T-scores (M = 50, SD = 10).

Academic achievement was measured using core subtests from either the Woodcock–Johnson Tests of Achievement, Third Edition (WJ III) [32] or Wechsler’s Individual Achievement Tests, Third Edition (WIAT-III) [33]. Eighty percent of the sample completed the WJ-III. Three subtests of the WJ-III were used for data analysis: Spelling, Calculation (to measure mathematics skills), and Letter-Word Identification (to measure reading skills). Similarly, three subtests of the WIAT-III were used for data analysis: Spelling, Numerical Operations (to measure mathematics skills), and Word Reading (to measure reading skills). All academic tasks were scored according to the guidelines in the test manual, and standard scores (mean of 100, standard deviation of 15) were derived using the published normative data which is adjusted for the child’s current grade level. The three subject area scores were averaged to create an “Academic Composite”, which was used in all analyses in an effort to measure academic achievement more broadly.

Though data from two academic tests was used, the tasks from both academic test batteries are comparable in scope and item content. That is, both spelling subtests require participants to dictate orally presented words that increase gradually in difficulty, both mathematics subtests require participants to solve mathematical equations that increase gradually in difficulty, and both reading subtests require participants to smoothly, orally decode English words that also increase gradually in difficulty. Normative values for both tests were established using large reference groups that were representative of the United States population across a variety of variables. Additionally, test developers for both test batteries correlated these measures against each other when determining convergent validity, which were noted to be high [32,33]. Thus, it was inferred that the subtests of interest on the WJ-III and WIAT-III assess the same underlying constructs and that scores could be used interchangeably.

Covariates were selected a priori based on theoretical support for their relevance in associations of interest. Gender and SES were included as covariates due to literature suggesting that they may modify the relationship between academic self-concept and academic performance [9,34]. Prior grade retention was selected as a covariate as it creates a shift in social comparison group while also providing significant negative feedback regarding the child’s academic competency [8]. Finally, primary diagnosis of a mood disorder was included as a covariate given that cognitive changes and decreased self-concept are both symptoms of depressive mood disorders. Of note, age was not included in the model because age-based, norm-referenced t-scores were utilized for the executive functioning and academic self-concept measures. The overall mediation model, as well as follow up analyses exploring potential interactive effects, was conducted using the PROCESS Macro for SPSS [35].

## 3. Results

Descriptive statistics were calculated and are reported in Table 3. As a whole, the sample demonstrated average academic self-concept and academic skills and clinically significant difficulties with practical executive functioning behaviors. Significant threats to univariate normality were observed for the BRIEF GEC and Academic Composite. As such, bias-corrected bootstrapping (1000 cases) was conducted for all analyses and bootstrapped standard errors were utilized in significance testing. Further, correlations between variables included in analyses were conducted to assess for multicollinearity (Table 4), and variables were not found to be highly correlated.

Analyses indicated a significant direct effect of practical executive functioning behaviors upon academic self-concept such that individuals who had more difficulty with executive functioning also had more problems with academic self-concept. However, this relationship was not significantly mediated by academic performance. In fact, academic skills were not significantly associated with problems with practical executive functioning behaviors or academic self-concept. In terms of covariates, significantly greater difficulty with academic self-concept was seen in girls. Overall, the final model accounted for 10% of the variance in academic self-concept (see Table 5).

Exploratory analyses were conducted to examine the moderating effect of gender on the association between difficulties with practical executive functioning behaviors and academic self-concept. However, while the association between executive dysfunction and academic self-concept was only significant in girls, the interaction between gender and problems with executive function was not significant (See Table 6).

## 4. Discussion

The results indicate that parent-reported difficulties with practical executive functioning behaviors are significantly associated with lower youth academic self-concept. However, neither of these variables was significantly associated with academic achievement testing measures. Furthermore, the current findings did not support moderation of the association between difficulties with practical executive functioning behaviors and academic self-concept by gender.

The findings suggest that difficulty with practical executive functioning behaviors may not significantly impact a young person’s ability to demonstrate academic achievement in a formal testing situation. However, there was an observed relationship between problems with executive functioning and self-perceived ability to accomplish goals and be academically successful (as measured by youth self-report on the BASC-2 Sense of Inadequacy scale). Additionally, the current results suggest that moderation of this association by gender is likely not strong enough to be of clinical utility.

The lack of association between problems with executive functioning and academic achievement in this sample is inconsistent with previous literature, which suggested that more problems with executive functioning would be associated with lower academic achievement [20,21,22,23,24]. However, this inconsistency may be explained by methodological and theoretical differences with the current study’s design. That is, previous research has assessed this association using performance-based measures for both executive functioning and academic constructs while the current study utilized parent-report measures to assess difficulties with practical executive functioning behaviors as they occur in everyday life. While performance-based measures are typically administered in a one-on-one setting, in an environment free from distractors, parent-report measures are designed to assess impairment that arises under everyday circumstances. Consistent with research indicating poor correlations between parent-report and performance-based executive functioning measures, the current findings may serve to highlight the notion that different measurement methods of executive functioning may have differing relationships with constructs of interest [18]. That is, though research indicates that performance-based executive functioning is associated with performance-based measures of academic achievement, ratings of problems with executive functioning may be more closely related to real-world measures of academic achievement, such as grade-point average. To directly assess this difference, future studies should simultaneously examine associations between differing measures of both executive functioning and other variables of interest, such as academic achievement.

Additionally, the specific academic subtests that were chosen for the current study were intended to assess mastery of concepts but did not require speeded performance nor integration of higher-order academic skills. Prior research in younger children suggests that this assessment of academic concepts, particularly in reading, may have tapped into a more crystallized or automatized process that did not require engagement of executive function for success [36]. Academic tests requiring youth to read and comprehend passages, construct essays, and solve mathematical word problems may be better measures of tasks difficult for youth with problems with executive function in the classroom. Thus, future studies should include these more integrative academic tasks that may be a better proxy for real-world academic achievement.

Further, the current findings are inconsistent with prior literature suggesting a significant positive association between ratings of emotion regulation and aspects of academic achievement, such as early performance-based reading and math skills and middle school grade point average. There are several possible explanations for this difference of findings. The association between ratings of emotion regulation and grade point average was observed in a non-clinical sample, which may have impacted generalizability to samples with significant impairments [26]. As noted, the utilization of grade point average as a measure of academic achievement may have also impacted findings, as ratings of executive functioning may be more closely related to every day classroom performance than to academic achievement as observed in a formal testing session. The association between ratings of emotion regulation and performance-based reading and mathematics was observed in a non-clinical sample of kindergarteners [25]. Thus, inconsistencies in findings may be related to developmental changes in these associations or differences between clinical and non-clinical populations.

Other theoretical conceptualizations of executive function may also explain differences between the current study’s findings and prior research indicating positive associations between ratings of emotion regulation and performance-based academic achievement. More specifically, it has been argued in the literature that emotion regulation, though related to executive functioning, should be considered a separate construct [12,13]. A confirmatory factor analysis of the BRIEF provides support for an Emotion Regulation Index, which was introduced in the revised measure but is still included in the Global Executive Composite [37]. Future research examining the association between academic achievement and areas of executive functioning should include domain-specific examination, especially when emotion regulation is included as a domain of executive function. Similarly, the current study did not find a significant association between academic achievement and self-concept. However, previous research has indicated an association between performance-based academic achievement and self-concept [6]. The social comparison aspect of self-concept indicates that youth judge their performance based on its comparison to that of the people around them [38]. This study’s sample consisted primarily of youth with ADHD, which is associated with executive skill deficits, at ages when typically developing individuals are rapidly acquiring executive skills (i.e., late childhood through adolescence), and problems with executive functioning were prominent in this sample. However, our participants’ academic achievement was average overall. Thus, it is possible that the basic academic skills measured in this study may have maintained stationary in regards to their impact on participants’ ability ranking compared to peers. In contrast, because executive functions develop rapidly in late childhood and adolescence, the widening executive skill gap between these youth and their peers may make problems with executive functioning more salient at this age. These problems with executive functioning may prevent these youth from demonstrating academic achievement in the classroom by, for example, preventing them from turning in homework or bringing necessary materials to class. Measures of real-world academic success (i.e., grade point average) may be more strongly associated with academic self-concept than performance-based measures of academic achievement, especially in youth with impairments which may prevent them from demonstrating academic achievement in a real-world setting.

Overall, the model accounted for only 10% of the variance despite the inclusion of a number of child-specific covariates that have been previously demonstrated to correlate with academic self-concept. What this model did not include are contextual factors, such as academic environment and teacher behaviors, which have been found to play a role in academic self-concept [39]. Further, many of these youth likely receive some form of special education services. While specific special education placements (i.e., mainstream classroom versus self-contained special education classroom) have not been shown to result in overall differences in self-concept, individual students may react differently to the feedback that they require instruction in a special classroom or conversely to the social comparison of working alongside typically developing peers as an individual with a disability. Thus, future research would benefit from inclusion of information regarding classroom placement and receipt of special education services [40]. The low variance accounted for by our identified child-level constructs speaks to the importance of continued examination of social influences and their interactive effects with child-level variables upon children’s self-perceptions.

This is the first known investigation into the association between academic self-concept and problems with executive functioning in a clinical sample. It is also one of few studies examining the association between practical executive functioning behaviors and academic achievement. The selected sample’s age range allows for examination of youth who have reached an age at which they likely have the capability to comment on their self-concept and their typically developing peers are rapidly acquiring executive skills. Additionally, use of assessment measures commonly utilized in clinical practice maximizes the applicability of the current findings.

However, the current study is not without limitations. This study utilized a cross-sectional design. As a result, the current findings are useful for developing hypotheses for future research but do not provide evidence of a causal relationship. Thus, it is unclear to what extent the results of the current study reflect a causal or bidirectional relationship between mood and cognition. That is, participants may exhibit low self-concept associated with a depressive mood state that could also impact problems with executive functioning [41]. Though the current study included primary diagnosis of a mood disorder as a covariate, it did not control specifically for depressed mood, and it is unknown how many participants exhibited comorbid depressive disorders.

Additionally, utilizing a parent-report measure as an indicator of youth problems with executive functioning may have falsely strengthened the identified association with academic self-concept. Parent-report measures assess parent perceptions of youth functioning. These perceptions, if conveyed to the child, may directly impact self-concept. Thus, current findings may reflect the impact that parent perception of problems with executive function has on youth self-concept rather than the association between self-concept and executive functioning itself. To circumvent this confound, studies seeking to replicate these findings may utilize alternative methods of assessing practical executive functioning behaviors, such as observational coding systems that do not require report from important adults in the youth’s life.

Furthermore, as archival clinical data were utilized, this study was limited to the use of measures chosen for clinical, rather than research, purposes. Thus, academic achievement was assessed using both the WJ-III and the WIAT-III. Though the academic subtests from the two academic achievement tests used for the current study appear to be very similar, and correlations conducted for norming of the measures suggest that the two tests use the same methods to examine the same underlying constructs, it would have been ideal for academic achievement to have been assessed using a single standard measure. Additionally, inclusion of a performance-based measure of executive functioning would have strengthened this study. However, due to the varying ages and referral questions of our participants, a consistent performance-based measure of executive functioning was not utilized in their assessments and as such was not available for analysis.

Should future studies address these limitations and replicate the current study’s finding that practical executive functioning behaviors are significantly associated with youth academic self-concept, further research examining other possible mediators that may be more closely related to ratings of problems with executive functioning (such as report card grades and academic achievement tasks that require more executive skills) as well as moderators would be indicated. Additionally, as executive dysfunction is a primary deficit in goal-oriented behavior, it stands to reason that functional impairments would span various contexts, affecting multiple domains of self-concept. For example, executive functioning has also been associated with peer relations [42]. Therefore, associations between executive functioning and other domains of self-concept, as well as global self-concept, should be explored. Replication and extension of the findings of the current study may eventually provide support for the inclusion of executive skills training in interventions designed to improve youth self-concept.

## Figures and Tables

**Table 1:** Diagnostic composition of study sample.

Primary Diagnosis	Percent of Sample
ADHD	56.6%
Traumatic Brain Injury	9%
Cancer/Tumors	7.4%
Specific Learning Disorders	6.6%
Epilepsy	5.7%
Intellectual Disability	4.1%
Other CNS Medical Conditions	4.1%
Mood Disorders	2.5%
Other Psychiatric Disorders	4.1%

ADHD, Attention-Deficit/Hyperactivity Disorder; CNS, central nervous system.

**Table 2:** Participant demographic information.

Demographic of Interest	Characteristic	Percent of Sample	M (SD)	Range	Possible Range
Gender	Male	62.3%			
Race	White	83.1%			
	Black or African American	12%			
	Asian	0.8%			
	More than one	2.5%			
	Declined to report	1.8%			
Ethnicity	Hispanic or Latino	8.2%			
Caregiver Relationship	(Biological or Adoptive) Mother	58.1%			
	(Biological or Adoptive) Father	27.8%			
	Other	14.1%			
Participant Age	Age in years		11.8 (2.7)	8–17	8–17
Socioeconomic Status	Hollingshead Score		37.3 (17.6)	11–77	11–77

**Table 3:** Descriptive statistics of study variables of interest.

Construct	Measure	Mean (SD)	Median	Mode
Executive Functioning	Global Executive Composite of BRIEF *	65.6 (12.4)	67	67
Academic Skills	Academic Composite	95.5 (11.5)	97.5	102
	Letter Word ID/Word Reading **	97.1 (12.9)	98	98
	Calculation/Numerical Operation **	95.4 (15.7)	96	97
	Spelling **	94.4 (14.5)	94	93
Academic Self-Concept	Sense of Inadequacy subscale of BASC-2 *	55.1 (12.1)	54	51

* T-Score (M = 50, SD = 10), ** Standard Score (M = 100, SD = 15). BRIEF, Behavioral Rating Inventory of Executive Functioning; BASC-2, Behavioral Assessment System for Children, Self-Report of Personality, Child and Adolescent versions, 2nd Edition.

**Table 4:** Correlation Matrix of Variables Included in Analyses.

Variable	Problems with Executive Function	Academic Achievement	Academic Self-Concept	SES	Gender	Primary Diagnosis of Mood Disorder
Academic Achievement	−0.16					
Academic Self-Concept	0.21 *	−0.06				
SES	0.29 *	−0.34 **	−0.06			
Gender	−0.02	−0.14	−0.19 *	0.12		
Primary Dianosis of Mood Disorder	0.05	0.08	0.19 *	0.12	−0.10	
Prior Grade Retention	0.14	−0.26 **	0.05	0.22 *	0.07	−0.13

Correlation is significant at * *p* < 0.05, ** *p* < 0.01. SES, socio-economic status.

**Table 5:** Model coefficients for mediational regression analysis.

Antecedent	Consequent					
	M (Academic Achievement)			Y (Academic Self-Concept)		
	Coeff.	*SE*	*p*	Coeff.	*SE*	*p*
X (Executive Functioning)	−0.06	0.08	0.5	0.18	0.09	0.04
M (Academic achievement)				−0.05	0.10	0.61
C_1_ (Prior Retention)	−4.22	2.06	0.04	0..92	2.23	0.23
C_2_ (Gender)	−2.28	2.03	0.26	−4.92	2.24	0.03
C_3_ (Primary Mood Disorder)	1.60	6.40	0.80	10.11	7.06	0.15
C_4_ (SES)	−0.17	0.06	<0.01	0.01	0.07	0.87
Constant	110.87	6.40	<0.001	54.	13.20	<0.001
	*R*^2^ = 0.16, *F*(5,116) = 2.92, *p* < 0.001			*R*^2^ = 0.10, *F*(6,115) = 2.19, *p* = 0.048		

**Table 6:** Moderated regression analysis examining the effect of the interaction between gender and problems with executive functioning on academic self-concept.

	Coeff.	*SE*	*p*
Problems with Executive Function	0.49	0.31	0.11
Gender	−7.45	12.13	0.54
Problems with Executive Function x Gender	−0.19	0.18	0.30
Prior Retention	1.08	2.23	0.64
Primary Mood Disorder Diagnosis	9.67	7.07	0.14
SES	0.00	0.07	0.95
Academic Achievement	−0.06	0.10	0.58
	*R*^2^ = 0.11, *F*(7,115) = 2.03, *p* = 0.06		

SE, standard error.
